# Localization of Stereovision for Measuring In-Crash Toeboard Deformation

**DOI:** 10.3390/s22082962

**Published:** 2022-04-12

**Authors:** Wei Zhang, Tomonari Furukawa, Azusa Nakata, Toru Hashimoto

**Affiliations:** 1 Department of Mechanical Engineering, University of Virginia, Charlottesville, VA 22903, USA; tomonari@virginia.edu; 2 Monozukuri Center, Automobile Operations, Honda Motor Co., Ltd., Haga-gun, Tochigi 321-3322, Japan; azusa_nakata@jp.honda (A.N.); toru_hashimoto@jp.honda (T.H.)

**Keywords:** toeboard deformation measurement, localization, recursive estimation, crash test, in-crash measurement

## Abstract

This paper presents a technique to localize a stereo camera for in-crash toeboard deformation measurement. The proposed technique designed a sensor suite to install not only the stereo camera but also initial measurement units (IMUs) and a camera for localizing purpose. The pose of the stereo camera is recursively estimated using the measurement of IMUs and the localization camera through an extended Kalman filter. The performance of the proposed approach was first investigated in a stepwise manner and then tested in controlled environments including an actual vehicle crash test, which had successfully resulted in measuring the toeboard deformation during a crash. With the oscillation motion in the occurrence of the crash captured, the deformation of the toeboard measured by stereo cameras can be described in a fixed coordinate system.

## 1. Introduction

The incidence of vehicle crashes keeps increasing with the production of vehicles, and more than six million crashes are reported every year these days in the United States, about thirty percent of which come with fatality and injury [1]. Vehicle crash is a severe and complicated dynamic phenomenon due to the complexity of physical interaction between the structures resulting from the impact. It is indispensable that the vehicles be crash-tested in indoor facilities and that the test results be used to design vehicles with improved crashworthiness [2,3,4,5,6]. In the crash tests, one of the focuses is laid on the toeboard deformation [7,8], which is due to the strong correlation between toeboard intrusion and lower extremity injuries, as seen both from collision statistics [9,10] and crash simulation [11]. A detailed analysis of toeboard deformation during a crash test can disclose useful intricacies about the otherwise unknown effect of the crash phenomenon.

In general, past work on vehicle deformation measurement can be classified into two approaches. In the first approach, finite element analysis (FEA) and other computational mechanics analyses have been used to predict instead of measuring the deformation. The advantage of numerical analysis is its ability to simulate all types of crash tests ([12,13,14,15]). Hickey and Xiao [16] used the three-dimensional (3D) model of a commercial vehicle and performed FEA to examine the effects of the deformation during a car crash test. Employing the full-scale 3D model directly for crash analysis and subsequent computational design requires large computational loads. Yang et al. [17] utilized the response surface method for a complex and dynamic large deformation problem to accelerate the analysis and design process. Pre- and post-measurements from the actual vehicle crash tests were used by Cheng et al. [18] and McClenathan et al. [19] to provide boundary conditions for FEA. Zhang et al. [20] reported the reconstruction of the deformation of a vehicle in a crash accident using high-performance parallel computing to incorporate an advanced elastic–plastic model. While the FEA has grown to be ever more important, its ability to estimate detailed deformations such as toeboard deformation is significantly limited because of the presence of various computation errors and the lack of actual measurements.

The second approach is based on the direct measurement. In the approach, vision-based measurement became most popular because of the ability of the camera to capture information of a continuous field ([21,22,23,24,25]). Digital image correlation, originally proposed by Sutton et al. [26], projects an electronic speckle pattern to the specimen to derive the deformation of the specimen from the displacement of the image of the speckle pattern on the specimen, and it has been widely employed to measure small deformation such as structure strain [27], cracks identification [28,29], or vibration of structures [30,31], in a static environment. Schmidt et al. [32] employed a stereo vision-based technique to measure the time-varying deformation of specimens in gas gun impact tests. As an alternative to the digital image correlation (DIC), Iliopoulos and Mchopoulos [33] developed the mesh-free random grid technique to measure deformation using marked dots, which was later referred to as Dot Centroid Tracking (DCT) for comparison with the DIC [34]. These techniques observed the deformation of a surface fixed to a static base from a static viewpoint. In general, while deformation measurement was well studied, the techniques cannot be applied to toeboard deformation measurement directly because of the oscillatory motion of the cameras. Since the line of sight of the toeboard is significantly limited, the cameras must be fixed near the toeboard to some part of the vehicle body, which deforms and oscillates by the crash. The measurement of toeboard through a stereo camera is therefore subject to the relative motion of the camera itself.

This paper presents a technique to localize a stereo camera for in-crash toeboard deformation measurement and a design to implement the technique in vehicle crash tests using a recursive state estimation [35,36,37]. This state estimation technique further completes our previous work that localizes the sensor suite using only the camera pose obtained through a computer vision technique [7], as the excessive oscillation of the localization camera due to the non-rigidity of the sensor suite poses extra errors in the measured toeboard deformation. In addition to the cameras for deformation measurement and localization, the proposed technique uses additional inertial measurement units (IMUs) for the localization. Having all the sensors in a rigid structure, the proposed technique implements extended Kalman filter (EKF) and estimates the pose of the stereo camera in the global coordinate frame using the observations of the downward camera and IMUs. In-crash toeboard deformation can thus be measured by subtracting the estimated stereo camera pose from the observed toeboard deformation. A sensor suite comprising the stereo camera, downward camera, and the IMUs is designed and placed on the seat mount such that the rigid body assumption is valid. The downward camera is located underneath the vehicle floor by making a hole.

This paper is organized as follows. The next section defines the state estimation problem of concern. Section 3 presents the proposed EKF-based technique to localize the stereo camera for the in-crash toeboard deformation measurement. Section 4 introduces the hardware design for our approach. The experimental results are then presented in Section 5. Conclusions and ongoing work are summarized in Section 6.

## 2. Sensor Suite and Localization Problem of Stereovision

### 2.1. Problem Formulation

**Figure 1:**
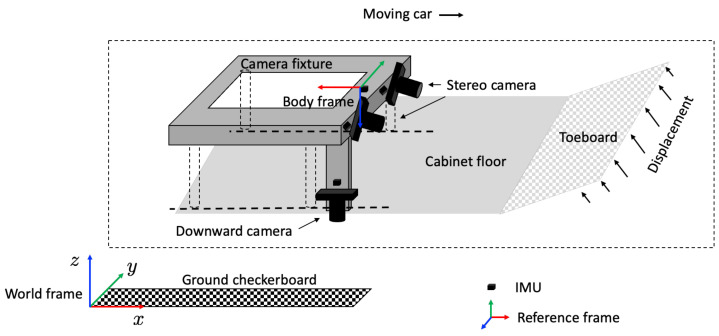
Sensor suite and localization problem formulation.

Figure 1 illustrates the fundamental design of the sensor suite adopted in this paper and the major components that address the problem of localizing the stereo camera. The stereo camera is fixed to the camera fixture, which is assumed to be rigid, and located such that it sees the toeboard. Additionally fixed to the camera fixture are the downward camera and the IMUs adjacent to the cameras, and a checkerboard is taped to the floor to localize the downward camera. The IMUs each consist of a triaxial gyroscope and an accelerometer. While the stereo camera measures the toeboard deformation in its camera coordinate frame, the downward camera and the IMUs are the sensors available to localize the pose of the stereo camera. The stereovision localization problem is thus defined as identifying the stereo camera pose given the images of the downward camera and the readings of the IMUs.

### 2.2. Linear Acceleration of Sensor Suite

In accordance with the rigid body dynamics, the acceleration (a) at the positions of IMUs is related to that of the center of the sensor suite by

(1) $$a=a_{c}+\dot{\omega }\times r+\omega \times \left(\omega \times r\right),$$

where $a_{c}$ is the acceleration of the center of the sensor suite body, ω is the angular velocity of the sensor suite body frame, and r is the relative position of the IMU to the center.

Note that it is more convenient to describe the acceleration relation (1) in the body frame, and that the cross-product identity $U\left(v_{1}\times v_{2}\right)=\left(Uv_{1}\right)\times \left(Uv_{2}\right)$ holds for unitary matrix $U\in R^{3\times 3}$. By left multiplying the transformation matrix from the inertial frame to the body frame, the acceleration relation (1) can be rewritten as

(2) $$a^{B}=a_{c}^{B}+{\dot{\omega }}^{B}\times r^{B}+\omega^{B}\times \left(\omega^{B}\times r^{B}\right),$$

where $a^{B}$, ${\dot{\omega }}^{B}$, $\omega^{B}$ and $r^{B}$ are the linear acceleration, angular acceleration, angular velocity, and position in the body frame (${}^{B}$).

**Figure 2:**
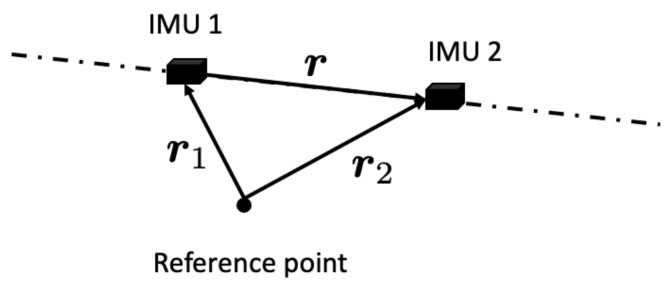
Illustration for angular acceleration using two IMUs.

The linear accelerations at different position of a rigid body need the input of angular acceleration. The input can be derived from the measurements of the triaxial accelerometers and gyroscopes in the IMUs. Because the derivation of angular acceleration at a point requires two IMU measurements, consider two IMUs in addition to the reference point as indicated in Figure 2. From Equation (1), the difference of the angular acceleration of IMU 1 from that of IMU 2 is given by

(3) $$a_{2}-a_{1}=\dot{\omega }\times \left(r_{2}-r_{1}\right)+\frac{1}{2}\omega_{2}\times \left(\omega_{2}\times r_{2}\right)-\frac{1}{2}\omega_{1}\times \left(\omega_{1}\times r_{1}\right),$$

where $a_{1},\;a_{2},\;\omega_{1}$, and $\omega_{2}$ are the accelerations and angular velocities at IMU 1 and 2, respectively. $r_{1}$ and $r_{2}$ are each the vector from the reference point to them. The position of the reference point for each pair of IMUs is chosen to be the midpoint of the IMU positions: $-r_{1}=r_{2}=\frac{1}{2}r_{12}\equiv \frac{1}{2}r$. This is to minimize the complexity and the error of transformation. The substitution of the midpoint into Equation (3) yields

(4) $$C\left(r\right)\dot{\omega }=b\equiv a_{1}-a_{2}+\frac{1}{2}\omega_{2}\times \left(\omega_{2}\times r\right)+\frac{1}{2}\omega_{1}\times \left(\omega_{1}\times r\right),$$

where the skew-symmetric *cross-product matrix* $C\left(r\right):R^{3}\to R^{3\times 3}$ is defined as

(5) $$C\left(r\right)=\left(\begin{array}{ccc} 0 & -r_{3} & r_{2}\\ r_{3} & 0 & -r_{1}\\ -r_{2} & r_{1} & 0 \end{array}\right),$$

where $r={\left(r_{1},r_{2},r_{3}\right)}^{T}$. The angular acceleration $\dot{\omega }$ can be computed through the IMU measurements of $a_{1}$, $a_{2}$, $\omega_{1}$, and $\omega_{2}$ by solving the linear problem (4).

### 2.3. Localization of Downward Camera

**Figure 3:**
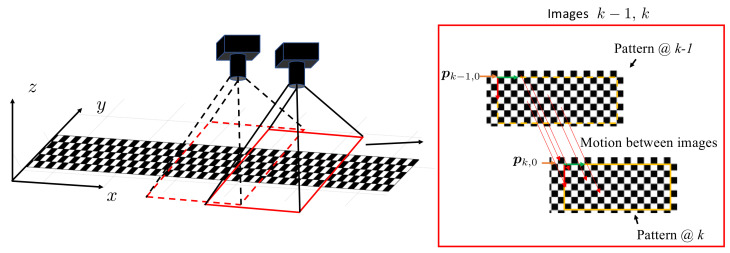
Scheme for downward camera localization.

The installation of the downward camera and the checkerboard on the ground is because the ground is the closest static surrounding structure to the sensor suite and thus can be measured most accurately. As illustrated in Figure 3, the global frame can be set at one end of the checkerboard and used to localize the downward camera. The downward camera observes the checkerboard pattern and any other extractable features. By associating the pattern and features between the global frame and pixel frame, the pose of the downward camera with respect to the global frame can be identified after calibrating the camera parameters following the procedures proposed by Zhang [38] or Heikkilä and Silvén [39]. In particular, for the checkerboard attached to the ground, the checkerboard corner points are readily identified [40] and serve as the planar pattern for the camera pose measurement, as illustrated in Figure 3.

For the global localization of the downward camera, let the pixel coordinates of the corner point (i,j) at the *k*th time step be $p_{k,ij}$. The pixel coordinates can be related to the corner point in the global frame by

(6) $$p_{k,ij}\to \left[\left(\begin{array}{c} N_{k}\\ 0 \end{array}\right)+\left(\begin{array}{c} i\\ j\\ 0 \end{array}\right)\right]w,$$

where *w* is the width of tiles of the checkerboard. $N_{k}$ is the number of tiles of the origin of the detected checkerboard at the current step relative to the origin at the first step, which can be accumulated by

(7) $$N_{k}=N_{k-1}+\mathrm{Integer}\left(\frac{\Delta_{k}+p_{k,0}-p_{k-1,0}}{{\bar{\Delta }}_{tile}}\right),$$

where $\Delta_{k}$ is the motion between two images in the pixel coordinates, and $p_{k,0}$ is the origin of the detected checkerboard origin in the pixel coordinate in image *k*. The initial $N_{0}$ is ${\left(0,0\right)}^{T}$.

The design and sensor settings adopted in this paper allow the motion of the sensor suite to be predicted with respect to the body frame and the pose of the downward camera to be measured in the global frame. While the downward camera is fixed to the sensor suite and thus can measure the sensor suite pose through the kinematic transformation, the pose predicted by the motion model and the angular acceleration measurement would be different from the pose measured by the downward camera and the kinematic transformation. The primary reason is the dead-reckoning errors stemming from the motion prediction with angular acceleration measurements. In addition, the pose of the downward camera alone contains excessive oscillations because of the non-rigidity of the sensor frame in the crash test. The acceleration measurement is in general noisy, and its integration creates notable integration errors, which accumulate over time in the name of dead-reckoning errors. Since the pose of the downward camera can be accurately measured because of its close distance to the ground, it is essential to estimate the pose of the sensor suite or the stereo camera by integrating the motion prediction and the observation. The next section presents the localization of a stereo camera proposed and formulated in the framework of EKF.

## 3. EKF-Based Localization of Stereo Camera for Toeboard Measurement

### 3.1. Overview

**Figure 4:**
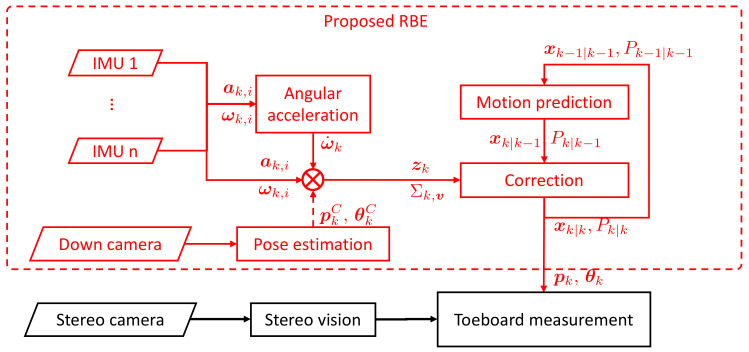
Proposed recursive localization of stereovision for in-crash toeboard measurement.

Figure 4 shows the EKF-based localization of the stereo camera proposed in this paper for toeboard deformation measurement. Since the camera fixture is assumed to be rigid and can transform the body frame to the stereo camera, to be identified are the position and orientation of the body frame, {p,θ}, where $p\in R^{3}$ is the position of the origin of the body frame and $\theta \in R^{3}$ is its Euler angle. To recursively estimate the motion of sensor suite using EKF framework, the state $x\equiv \{p,\;\dot{p},\;\ddot{p},\;\theta ,\;\omega^{B},\;{\dot{\omega }}^{B}\}$ includes not only the pose, but also velocity $\dot{p}$, acceleration $\ddot{p}$, angular velocity $\omega^{B}$, and angular acceleration ${\dot{\omega }}^{B}$ in the body frame.

In accordance with the EKF, the primary processes of estimation are the prediction and the correction. The prediction of the state at discrete time *k*, or the derivation of the mean $x_{k|k-1}$ and the covariance $P_{k|k-1}$, is performed using the motion model of a sensor suite and its state estimated at k−1, i.e., $x_{k-1|k-1}$ and $P_{k-1|k-1}$. In the correction step, the predicted sensor suite state is corrected to $x_{k|k}$ and $P_{k|k}$ by fusing the measurements of IMUs ($a_{k}$ and $\omega_{k}$), downward camera pose ($p_{k}^{C}$ and $\theta_{k}^{C}$), and angular acceleration (${\dot{\omega }}_{k}$) described in Section 2.2 and Section 2.3. While different sensors are used for the same state, Kalman gain is adjusted through the statistic property of motion and sensor noise via their covariance $P_{k|k-1}$ and $\Sigma_{k,v}$ and provides the optimal estimate for each state in the proposed approach. Once the pose of the sensor suite is globally estimated, the deformation of the toeboard can be measured with respect to the global and any other coordinate frames.

The operation of the EKF needs motion and sensor models. The discrete motion model of the sensor suite and the observation model subjected to uncertainty are generically given by

(8) $$x_{k}=f\left(x_{k-1},w_{k}\right),\;\mathrm{and}\;z_{k}=h\left(x_{k},v_{k}\right),$$

where f(·) and h(·) are the respective motion and sensor models; $w_{k}$ and $v_{k}$ represent the motion model noise and sensor model noise, respectively. The subsequent two sections present the motion and sensor models developed in the proposed approach.

### 3.2. Motion Model of Sensor Suite

The motion of the sensor suite pose is determined by the motion and the deformation of the entire vehicle, which cannot be modeled and identified easily. Meanwhile, the sensor suite motion is constrained by the motion and the deformation of the vehicle. This means that the range of the sensor suite motion is bounded. With a short time step Δt, the proposed approach accordingly predicts the pose of the sensor suite by the random walk motion model as

(9) $$\begin{array}{cc} p_{k} & =p_{k-1}+\Delta t\;{\dot{p}}_{k-1}+\frac{1}{2}\Delta t^{2}\left({\ddot{p}}_{k-1}+\epsilon_{k,\ddot{p}}\right),\\ {\dot{p}}_{k} & ={\dot{p}}_{k-1}+\Delta t\left({\dot{p}}_{k-1}+\epsilon_{k,\ddot{p}}\right),\\ {\ddot{p}}_{k} & ={\ddot{p}}_{k-1}+\epsilon_{k,\ddot{p}},\\ \theta_{k} & =\theta_{k-1}+\Delta tE\left(\theta_{k}\right)\left(\omega_{k-1}^{B}+\Delta t\left({\dot{\omega }}_{k-1}^{B}+\epsilon_{k,\dot{\omega }}\right)\right),\\ \omega_{k}^{B} & =\omega_{k-1}^{B}+\Delta t\left({\dot{\omega }}_{k-1}^{B}+\epsilon_{k,\dot{\omega }}\right),\\ {\dot{\omega }}_{k}^{B} & ={\dot{\omega }}_{k-1}^{B}+\epsilon_{k,\dot{\omega }}, \end{array}$$

where $\left\{\epsilon_{k,\ddot{p}},\epsilon_{k,\dot{\omega }}\right\}$ is a motion noise due to unresolved linear and angular acceleration, and $\theta ={\left[\phi ,\theta ,\varphi \right]}^{⊤}$ are the Euler angles corresponding to the roll, pitch, and yaw motion of the sensor suite. In the equation

(10) $$E\left(\theta \right)=\left(\begin{array}{ccc} 1 & \sin(\phi )\tan(\theta ) & \cos(\phi )\tan(\theta )\\ 0 & \cos(\phi ) & -\sin(\phi )\\ 0 & \sin(\phi )/\cos(\theta ) & \cos(\phi )/\cos(\theta ) \end{array}\right),$$

is the *Euler angle rates matrix* [41], whose multiplication with the body-fixed angular velocity yields Euler angle rates. Through linearization, the motion model can be encapsulated into a canonical form as:

(11) $$x_{k}=F_{k}x_{k-1}+V_{k}w_{k},$$

where $F_{k}$ and $V_{k}$ are the Jacobian matrix of the motion and the motion noise term, respectively, and are given by

(12) $$F_{k}=\left(\begin{array}{cc} F_{1} & 0_{9\times 9}\\ 0_{9\times 9} & F_{2,k} \end{array}\right),\;\mathrm{and}\;V_{k}=\left(\begin{array}{cc} V_{1} & 0_{9\times 3}\\ 0_{9\times 3} & V_{2,k} \end{array}\right),$$

where

$$\begin{array}{ccc} & F_{1}=\left(\begin{array}{ccc} I_{3} & \Delta tI_{3} & \frac{\Delta t^{2}}{2}I_{3}\\ 0_{3} & I_{3} & \Delta tI_{3}\\ 0_{3} & 0_{3} & I_{3} \end{array}\right), & F_{2,k}=\left(\begin{array}{ccc} I_{3} & \Delta tE(\theta_{k}) & \Delta t^{2}E\left(\theta_{k}\right)\\ 0_{3} & I_{3} & \Delta tI_{3}\\ 0_{3} & 0_{3} & I_{3} \end{array}\right),\\ & V_{1}=\left(\begin{array}{c} \frac{\Delta t^{2}}{2}I_{3}\\ \Delta tI_{3}\\ I_{3} \end{array}\right), & V_{2,k}=\left(\begin{array}{c} \Delta t^{2}E\left(\theta_{k}\right)\\ \Delta tI_{3}\\ I_{3} \end{array}\right). \end{array}$$

$w_{k}∼N\left(0,\Sigma_{k,w}\right)$ is a motion noise, which is validly assumed to be Gaussian since Δt is small.

### 3.3. Sensor Models for Localization of Sensor Suite

#### 3.3.1. Sensor Models of Accelerometer

According to Equation (2), the sensor model outputting linear acceleration with respect to the body frame can be approximated with Gaussian noise as

(13) $$\begin{array}{c} z_{k,a}=h_{k,a}^{\ddot{p}}\left(x_{k},r^{B}\right)+v_{a}\equiv R{\left(\theta_{k}\right)}^{T}{\ddot{p}}_{k}+{\dot{\omega }}_{k}^{B}\times r^{B}+\omega_{k}^{B}\times \left(\omega_{k}^{B}\times r^{B}\right)+v_{a}, \end{array}$$

where $z_{k,a}$ is the measurement of linear acceleration, $r^{B}$ is the position of accelerometer in the body frame, and $v_{a}∼N\left(0,\Sigma_{a}\right)$ is the measurement noise of the accelerometer. R(θ) is the *rotation matrix* [41] to transfer the body frame to the inertial frame.

Because of the association through integration, the measurement $z_{k,a}$ can also be represented with the velocity and the position. This additionally introduces two sensor models:

(14) $$\begin{array}{cc} z_{k,a} & =h_{k,a}^{p}\left(x_{k},r^{B}\right)+v_{a}\equiv R{\left(\theta_{k}\right)}^{T}\frac{p_{k}-2p_{k-1}+p_{k-2}}{\Delta t^{2}}+{\dot{\omega }}_{k}^{B}\times r^{B}+\omega_{k}^{B}\times \left(\omega_{k}^{B}\times r^{B}\right)+v_{a},\\ z_{k,a} & =h_{k,a}^{\dot{p}}\left(x_{k},r^{B}\right)+v_{a}\equiv R{\left(\theta_{k}\right)}^{T}\frac{{\dot{p}}_{k}-{\dot{p}}_{k-1}}{\Delta t}+{\dot{\omega }}_{k}^{B}\times r^{B}+\omega_{k}^{B}\times \left(\omega_{k}^{B}\times r^{B}\right)+v_{a}. \end{array}$$

Let the measurements and the sensor models be described collectively as

(15) $$z_{k}^{a}=\left(\begin{array}{c} z_{k,a}\\ z_{k,a}\\ z_{k,a} \end{array}\right),\;h_{k}^{a}\left(x_{k}\right)=\left(\begin{array}{c} h_{k,a}^{p}\\ h_{k,a}^{\dot{p}}\\ h_{k,a}^{\ddot{p}} \end{array}\right).$$

The corresponding Jacobian matrix is written as

(16) $$H_{k,a}\equiv \frac{\partial h_{k}^{a}}{\partial x}=\left(\begin{array}{cccccc} \frac{R{\left(\theta \right)}^{T}}{\Delta t^{2}} & 0_{3} & 0_{3} & \frac{\partial h_{k,a}^{p}}{\partial \theta_{k}} & \frac{\partial h_{k,a}^{\ddot{p}}}{\partial \omega_{k}^{B}}-\frac{C(r^{B})}{\Delta t} & 0_{3}\\ 0_{3} & \frac{R{\left(\theta \right)}^{T}}{\Delta t} & 0_{3} & \frac{\partial h_{k,a}^{\dot{p}}}{\partial \theta_{k}} & \frac{\partial h_{k,a}^{\ddot{p}}}{\partial \omega_{k}^{B}}-\frac{C(r^{B})}{\Delta t} & 0_{3}\\ 0_{3} & 0_{3} & R{\left(\theta \right)}^{T} & \frac{\partial h_{k,a}^{\ddot{p}}}{\partial \theta_{k}} & \frac{\partial h_{k,a}^{\ddot{p}}}{\partial \omega_{k}^{B}} & -C(r^{B}) \end{array}\right),$$

where C(·) is the skew symmetric matrix in Equation (5) and

$$\begin{array}{cc} \frac{\partial h_{k,a}^{p}}{\partial \theta_{k}} & =\frac{\partial R{\left(\theta \right)}^{T}\frac{\delta^{2}p_{k}}{\Delta t^{2}}}{\partial \theta },\;\frac{\partial h_{k,a}^{\dot{p}}}{\partial \theta_{k}}=\frac{\partial R{\left(\theta \right)}^{T}\frac{\delta {\dot{p}}_{k}}{\Delta t}}{\partial \theta },\\ \frac{\partial h_{k,a}^{\ddot{p}}}{\partial \theta_{k}} & =\frac{\partial R{\left(\theta \right)}^{T}p_{k}}{\partial \theta },\\ \frac{\partial h_{k,a}^{\ddot{p}}}{\partial \omega_{k}^{B}} & =-C\left(\omega_{k}^{B}\times r^{B}\right)-C\left(\omega_{k}^{B}\right)C\left(r^{B}\right), \end{array}$$

where δ represents the finite difference operation and $\delta^{2}p_{k}=p_{k}-2p_{k-1}+p_{k-2}$ and $\delta {\dot{p}}_{k}={\dot{p}}_{k}-{\dot{p}}_{k-1}$.

#### 3.3.2. Sensor Models of Gyroscope

Because the gyroscope measures the angular velocity, the proposed approach constructs two sensor models, each outputting the angular velocity and the sensor suite orientation:

(17) $$\begin{array}{cc} z_{k,g} & =h_{k,g}^{\omega }\left(x_{k},v\right)\equiv \omega_{k}^{B}+v_{g}\;,\\ z_{k,g} & =h_{k,g}^{\theta }\left(x_{k},v\right)\equiv E{\left(\theta_{k}\right)}^{-1}\frac{\theta_{k}-\theta_{k-1}}{\Delta t}+v_{g}\;, \end{array}$$

where $v_{g}$ is the gyroscope measurement noise, and the inverse of the Euler angle rates matrix is given by

$$E{\left(\theta \right)}^{-1}=\left(\begin{array}{ccc} 1 & 0 & -\sin\theta \\ 0 & \cos\phi & \sin\phi \cos\theta \\ 0 & -\sin\phi & \cos\phi \cos\theta \end{array}\right).$$

Let the measurements and the sensor models be described collectively again:

(18) $$z_{k}^{g}=\left(\begin{array}{c} z_{k,g}\\ z_{k,g} \end{array}\right),\;h_{k}^{g}\left(x_{k}\right)=\left(\begin{array}{c} h_{k,g}^{\theta }\\ h_{k,g}^{\omega } \end{array}\right).$$

The Jacobian matrix is given by

(19) $$H_{k,g}\equiv \frac{\partial h_{k}^{g}}{\partial x}=\left(\begin{array}{cccccc} 0_{3} & 0_{3} & 0_{3} & \frac{E^{\prime }\left(\delta \theta_{k}\right)+E{\left(\theta_{k}\right)}^{-1}}{\Delta t} & 0_{3} & 0_{3}\\ 0_{3} & 0_{3} & 0_{3} & 0_{3} & I_{3} & 0_{3} \end{array}\right),$$

with

(20) $$E^{\prime }\left(v\right)=\left(\begin{array}{ccc} 0 & -v_{3}\cos\theta & 0\\ -v_{2}\sin\phi +v_{3}\cos\phi \cos\theta & -v_{3}\sin\phi \sin\theta & 0\\ -v_{2}\cos\phi -v_{3}\sin\phi \cos\theta & -v_{3}\cos\phi \sin\theta & 0 \end{array}\right).$$

$\delta \theta_{k}=\theta_{k}-\theta_{k-1}$ is the finite difference.

#### 3.3.3. Sensor Models for Angular Acceleration

The derivation of the angular acceleration ${\dot{\omega }}_{k}^{B}$ in Equation (13) from accelerometers in Equations (3)–(5) additionally introduces sensor models for angular acceleration. They are associated with the angular acceleration, the angular velocity, and the orientation as

(21) $$\begin{array}{cc} z_{k,\dot{\omega }} & =h_{k,\dot{\omega }}^{\dot{\omega }}\left(x\right)\equiv {\dot{\omega }}_{k}^{B}+v_{k,\dot{\omega }},\\ z_{k,\dot{\omega }} & =h_{k,\dot{\omega }}^{\omega }\left(x\right)\equiv \frac{\omega_{k}^{B}-\omega_{k-1}^{B}}{\Delta t}+v_{k,\dot{\omega }},\\ z_{k,\dot{\omega }} & =h_{k,\dot{\omega }}^{\theta }\left(x\right)\equiv E{\left(\theta \right)}^{-1}\frac{\theta_{k}-2\theta_{k-1}+\theta_{k-2}}{\Delta t^{2}}+v_{k,\dot{\omega }}\;.\;\; \end{array}$$

where $v_{k,\dot{\omega }}∼N\left(0,\Sigma_{\dot{\omega }}\right)$ is a measurement noise for angular acceleration. Let the measurements and the sensor models be described collectively:

(22) $$z_{k}^{\dot{\omega }}=\left(\begin{array}{c} z_{k,\dot{\omega }}\\ z_{k,\dot{\omega }}\\ z_{k,\dot{\omega }} \end{array}\right),\;h_{k}^{\dot{\omega }}\left(x_{k}\right)=\left(\begin{array}{c} h_{k,\dot{\omega }}^{\theta }\\ h_{k,\dot{\omega }}^{\omega }\\ h_{k,\dot{\omega }}^{\dot{\omega }} \end{array}\right).$$

The Jacobian matrix is obtained by

(23) $$H_{k,\dot{\omega }}\equiv \frac{\partial h_{k}^{\dot{\omega }}\left(x_{k}\right)}{\partial x}=\left(\begin{array}{cccccc} 0_{3} & 0_{3} & 0_{3} & \frac{E^{\prime }\left(\delta^{2}\theta_{k}\right)+E{\left(\theta_{k}\right)}^{-1}}{\Delta t^{2}} & 0_{3} & 0_{3}\\ 0_{3} & 0_{3} & 0_{3} & 0_{3} & \frac{I_{3}}{\Delta t} & 0_{3}\\ 0_{3} & 0_{3} & 0_{3} & 0_{3} & 0_{3} & I_{3} \end{array}\right)$$

with $E^{\prime }\left(\cdot \right)$ being defined in Equation (20) and $\delta^{2}\theta_{k}=\theta_{k}-2\theta_{k-1}+\theta_{k-2}$.

While the sensor models have been constructed, the remaining modeling process for angular acceleration is the derivation of the covariance $\Sigma_{\dot{\omega }}$ for angular acceleration from a pair of IMUs. We expand the right-hand side of Equation (4) by considering noise on top of the measurement of acceleration and angular velocity:

$$\begin{array}{cc} b+v_{b}= & -\left(a_{2}-a_{1}\right)+\frac{1}{2}\omega_{2}\times \left(\omega_{2}\times r\right)+\frac{1}{2}\omega_{1}\times \left(\omega_{1}\times r\right)\\ \; & +v_{a_{2}}+\frac{1}{2}\omega_{2}\times \left(v_{\omega_{2}}\times r\right)+\frac{1}{2}v_{\omega_{2}}\times \left(\omega_{2}\times r\right)\\ \; & +v_{a_{1}}+\frac{1}{2}\omega_{1}\times \left(v_{\omega_{1}}\times r\right)+\frac{1}{2}v_{\omega_{1}}\times \left(\omega_{1}\times r\right)\\ \; & +\frac{1}{2}v_{\omega_{2}}\times \left(v_{\omega_{2}}\times r\right)+\frac{1}{2}v_{\omega_{1}}\times \left(v_{\omega_{1}}\times r\right)\;, \end{array}$$

where $v_{b}$ is the noise on vector b, $v_{a_{1}}$ and $v_{a_{1}}$ are the measurement noise of the accelerometer, and $v_{\omega_{1}}$ and $v_{\omega_{2}}$ are that of the gyroscope. By neglecting the smaller quadratic noise terms above, the covariance can be computed as

(24) $$\Sigma_{b}\approx \Sigma_{a_{1}}+\Sigma_{a_{2}}+\frac{1}{2}{\tilde{C}}_{1}\Sigma_{\omega_{1}}{\tilde{C}}_{1}^{T}+\frac{1}{2}{\tilde{C}}_{2}\Sigma_{\omega_{2}}{\tilde{C}}_{2}^{T},$$

where

$$\begin{array}{cc} {\tilde{C}}_{1} & =C\left(\omega_{1}\times r\right)+C\left(\omega_{1}\right)C\left(r\right),\\ {\tilde{C}}_{2} & =C\left(\omega_{2}\times r\right)+C\left(\omega_{2}\right)C\left(r\right). \end{array}$$

C(·) is the cross-product matrix defined in Equation (5).

#### 3.3.4. Sensor Model of Downward Camera

Since the downward camera ultimately derives its pose with respect to the global frame, the sensor model associates the observations with the pose of the sensor suite:

(25) $$\begin{array}{cc} z_{k,p} & =h_{k,p}\left(x_{k},v_{k,p}\right)\equiv p_{k}+R{\left(\theta_{k}\right)}^{T}r_{c}^{B}+v_{k,p},\\ z_{k,\theta } & =h_{k,\theta }\left(x_{k},v_{k,\theta }\right)\equiv \theta_{k}+v_{k,\theta }, \end{array}$$

where $r_{c}^{B}$ is the coordinate of the downward camera in the body frame. Let the measurements and the sensor models be described collectively:

(26) $$z_{k,c}=\left(\begin{array}{c} p_{k,c}\\ \theta_{k,c} \end{array}\right),\;\;h_{k,c}\left(x_{k}\right)\equiv \left(\begin{array}{c} p_{k}+R{\left(\theta_{k}\right)}^{T}r_{c}^{B}\\ \theta_{k} \end{array}\right)+v_{k,c}\;,$$

The Jacobian of the downward camera model is given by

(27) $$H_{k,c}\equiv \frac{\partial h_{k,c}}{\partial x}=\left(\begin{array}{cccccc} I_{3} & 0_{3} & 0_{3} & \frac{\partial R{\left(\theta \right)}^{T}r_{c}^{B}}{\partial \theta } & 0_{3} & 0_{3}\\ 0_{3} & 0_{3} & 0_{3} & I_{3} & 0_{3} & 0_{3} \end{array}\right).$$

### 3.4. EKF

The implementation of the EKF for the localization of the sensor suite is relatively straightforward once the motion model and all the sensor models have been developed. Given the motion model (11), the proposed approach predicts the mean and the covariance, $x_{k|k-1}$ and $P_{k|k-1}$, using its prior belief $x_{k-1|k-1}$ and $P_{k-1|k-1}$ as

(28) $$\begin{array}{cc} x_{k|k-1} & =F_{k}x_{k-1|k-1},\\ P_{k|k-1} & =F_{k}P_{k-1|k-1}F_{k}^{⊤}+V_{k}\Sigma_{k,w}V_{k}^{⊤}. \end{array}$$

The sensor models are each given by a function of some state variables. Accordingly, the proposed approach corrects the estimation through the parallel KF where the correction of each state from each sensor is fused through summation [42]:

(29) $$\begin{array}{cc} x_{k|k} & =x_{k|k-1}+\sum_{s\in S}K_{k,s}\left(z_{k,s}-h_{k,s}\left(x_{k|k-1}\right)\right),\\ P_{k|k}^{-1} & =P_{k|k-1}^{-1}+\sum_{s\in S}H_{k,s}^{T}\Sigma_{k,s}^{-1}H_{k,s}\;, \end{array}$$

where s∈S is each sensor, and $\Sigma_{k,s}$ and $H_{k,s}$ are the covariance of measurement noise of sensor and the Jacobian matrix of the sensor model $h_{k,s}\left(x\right)$, respectively. Kalman gain for sensor *s* is given by

(30) $$K_{k,s}=P_{k|k}H_{k,s}^{T}\Sigma_{k,s}^{-1}\;.$$

The great advantage of the proposed formulation is the simplicity. While the dimension of each correction is different, the Kalman gain acts not only as a scaling factor but also as a transformation matrix. All the correction terms are thus summed without loss of generality.

## 4. Sensor Suite Design and Installation

**Figure 5:**
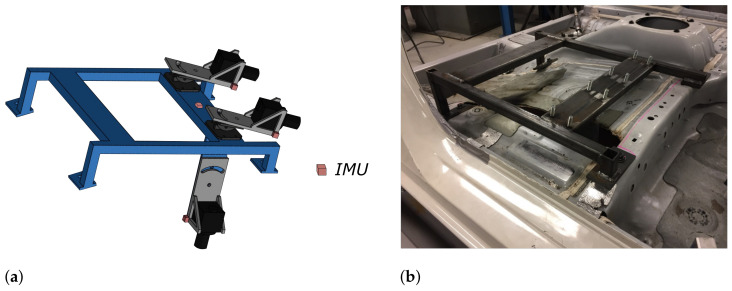
Sensor suite design (**a**) and installation (**b**).

Figure 5a shows the design of the sensor suite with all sensors installed. The stereo cameras are installed on the sensor suite using an orientation-adjustable base to have a better view of the toeboard. The downward camera is installed on a leg welded on the sensor suite. A three-axis gyroscope and a three-axis accelerometer are equipped on each camera to estimate its position and orientation. The frame size is 560mm×450mm×130mm. The size enables the sensor suite to be installed on the mounting bolt holes of the front seat without modifications to the test vehicle structure, as shown in Figure 5b.

The sensor specifications are shown in Table 1. Since the the crash of vehicle test typically lasts 100∼200 ms, high-speed sensors with a wide measurement range are selected.

**Figure 6:**
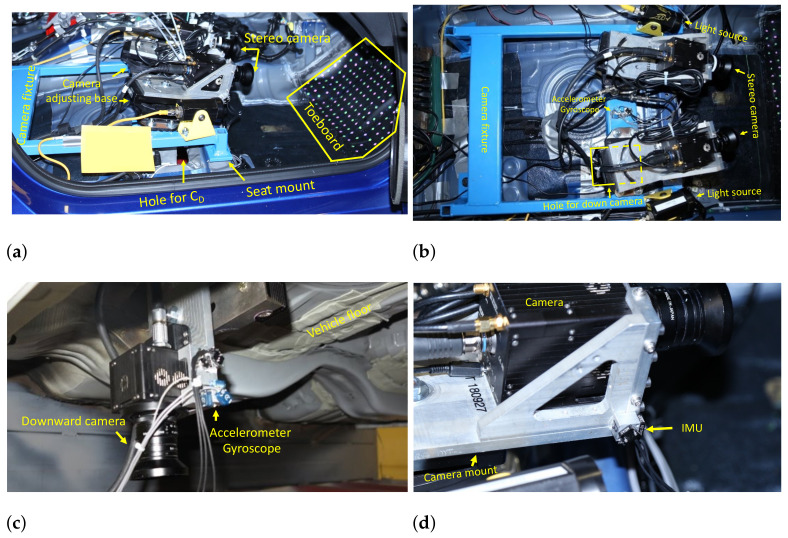
Sensor suite for real car test: (**a**) side view; (**b**) top view; (**c**) bottom view; (**d**) close-up view of camera and IMU sensors.

The sensor suite was designed and tested in a laboratory environment. It was later tested at the crash test center of Honda Research & Development Americas, Inc. (Ohio, USA), in a full-size passenger car. Figure 6a,b show the side and top view of the sensor suite installed on the test vehicle. The stereo camera was placed 400mm away from the toeboard so both cameras could have a full view of it. A hole with size 150mm×150mm was opened on the vehicle floor to install the downward camera, as shown in Figure 6b,c. The downward camera was about 95mm above the ground and had a clear view of the checkerboard pattern. Additional installations of IMU are shown in Figure 6c,d.

## 5. Experimental Results

This section first demonstrates the localization of the sensor suite using the proposed EKF framework in a simulated environment. Then, results of a real car crash test are followed.

### 5.1. Sensor Suite Localization in Simulated Environment

Consider the following case where the center of sensor suite undergoes simple linear and angular motion in the global frame as

$$\begin{array}{cc} a & ={\left(\begin{array}{ccc} -{\left(2\pi f\right)}^{2}\sin\left(2\pi ft\right), & 0, & 0 \end{array}\right)}^{T},\\ \omega & ={\left(\begin{array}{ccc} -2\pi f_{w}\left(\cos(2\pi f_{w}t)-1\right), & 0, & 0 \end{array}\right)}^{T}, \end{array}$$

with angular acceleration

$$\dot{\omega }={\left(\begin{array}{ccc} {\left(2\pi f_{w}\right)}^{2}\sin{\left(2\pi f_{w}t\right)}^{T}, & 0, & 0 \end{array}\right)}^{T}.$$

The measurement of the accelerometer and gyroscope in the body frame shall be

(31) $$\begin{array}{cc} a_{IMU}^{B} & =a_{c}^{B}+{\dot{\omega }}^{B}\times r^{B}+\omega^{B}\times \left(\omega^{B}\times r^{B}\right)+v_{a},\\ \omega^{B} & =-\omega +v_{g} \end{array}$$

where $a_{c}^{B}$ and ${\dot{\omega }}^{B}$ are measurements at sensor suite center as

$$a_{c}^{B}=-a,\;{\dot{\omega }}^{B}=-\dot{\omega }$$

and $v_{g}∼N\left(0,\sigma_{g}\right)$ and $v_{a}∼N\left(0,\sigma_{a}\right)$ model the sensor noise of accelerometer and gyroscope. The pose of downward camera shall be provided by

(32) $$\begin{array}{cc} p_{C_{D}} & =p+R\left(\theta \right)r_{C_{D}}+v_{c,p}\\ \theta_{C_{D}} & =\theta +v_{c,\theta }, \end{array}$$

where the position and orientation of the sensor suite are

$$\begin{array}{cc} p & ={\left(\begin{array}{ccc} \sin(2\pi ft)-2\pi ft-2\pi , & 0, & 0 \end{array}\right)}^{T},\\ \theta & ={\left(\begin{array}{ccc} -\sin\left(2\pi f_{w}t\right)+2\pi f_{w}t+2\pi , & 0, & 0 \end{array}\right)}^{T}. \end{array}$$

Here, R(θ) is the rotation matrix given Euler angle θ, and $r_{C_{D}}^{B}$ is the position of downward camera in the body frame. Sensor noise of camera $v_{c}∼N\left(0,\sigma_{c}\right)$ is Gaussian. IMU data are available between −0.1s∼0.3s while camera pose information is available between −0.01s∼0.2s, to be consistent with the real car crash test.

**Figure 7:**
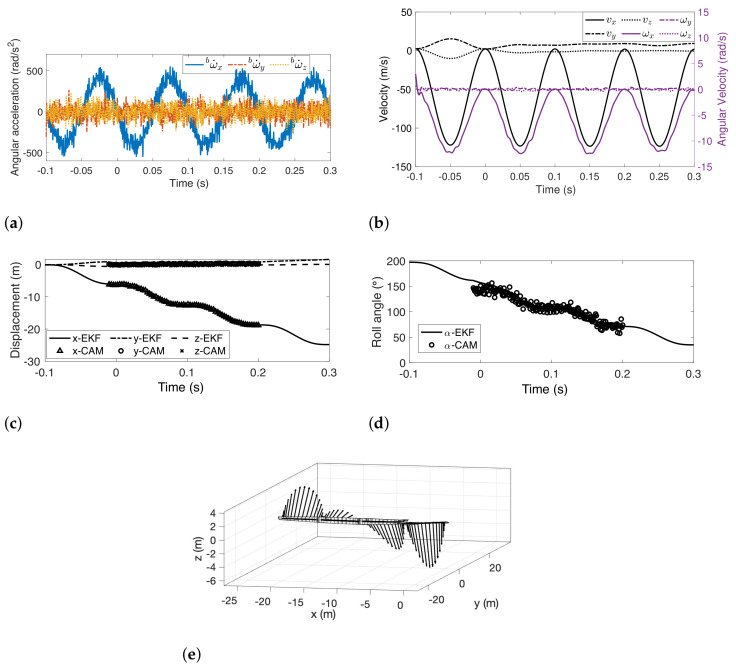
Results of sensor suite localization using EKF framework in a simulated environment. (**a**) Angular acceleration; (**b**) linear and angular velocity of the camera fixture; (**c**) Estimated displacement of the camera fixture; (**d**) estimated roll angle of the camera fixture; (**e**) trajectory of the camera fixture in space.

By letting $f=f_{w}=10$, the angular acceleration can be recovered from the measurement of accelerometers and gyroscope, as illustrated in Figure 7a. This angular acceleration follows the prescribed ${\dot{\omega }}^{B}$. The amplitude in the x-direction is close to the given amplitude ${\left(2\pi f_{w}\right)}^{2}$. The other two directions (${\dot{\omega }}_{y}$ and ${\dot{\omega }}_{z}$) are zero-meaned. The estimated velocity is shown in Figure 7b. Linear velocity and angular velocity at x-direction follow the cosine wave, while motion in other directions is trivial. The displacement and roll angles are shown in Figure 7c,d. Figure 7e illustrates the motion of the sensor suite in space, where rectangular panels illustrate the position with a normal vector for the orientation.

**Figure 8:**
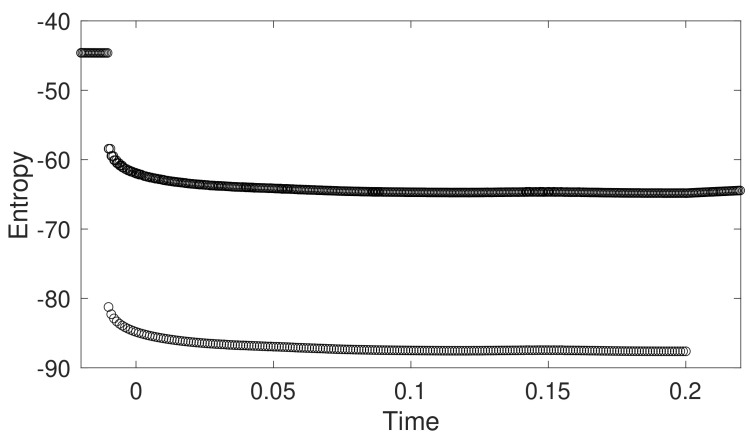
Entropy of sensor suite localization in simulated environment.

Differential entropy of the estimation is plotted in Figure 8, which is given by

$$h\left(x\right)=\frac{1}{2}\log\left[{\left(2\pi e\right)}^{n}\left|\Sigma_{k|k}\right|\right]$$

for multivariate Gaussian distribution considered here. The entropy shows that the downward camera pose significantly improves the certainty of the estimation, as shown in Figure 8.

**Figure 9:**
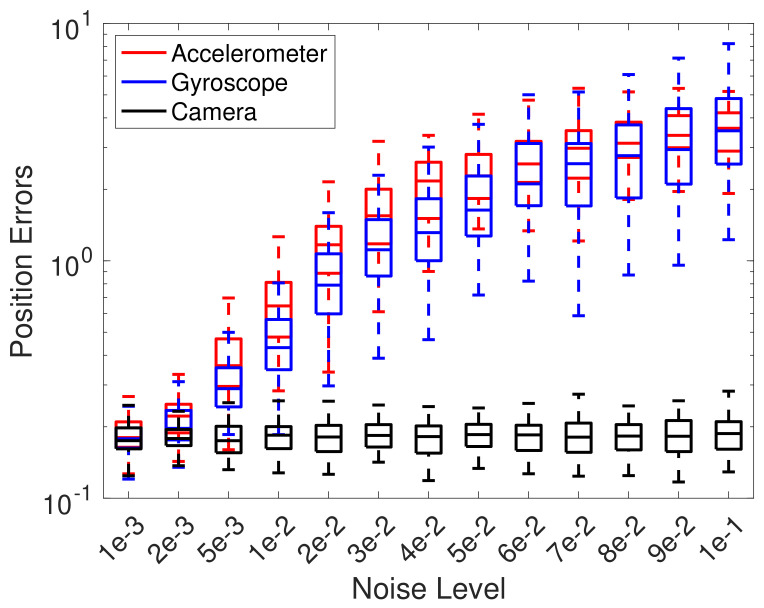
Error of EKF estimation under different noise level on accelerometer, gyroscope, and camera measurement.

To test the limits of the proposed EKF framework, we vary noise level by $\sigma_{a}$, $\sigma_{g}$, or $\sigma_{c}$ individually while keeping the other two at a low level, 0.1% of the maximum value, to be specific. For each combination, 100 tests were performed and the statistics of maximum error of the position $\max∥p_{k|k}-p_{k}∥$ are plotted in Figure 9, where $p_{k|k}$ is the estimation and $p_{k}$ is the ground truth at step *k*. The figure shows that the proposed EKF-based estimation is accurate when noise is typically small, say, less than 1%. Then, error can grow significantly when the noise of accelerometers and gyroscope further increases. Note that dead-reckoning error comes from measurements of accelerometer and gyroscope; therefore, noise on the downward camera pose may not significantly influence the estimation if IMU measurements are not significantly noisy.

### 5.2. Sensor Suite Localization in 56 km/h Frontal Barrier Car Crash Test

#### 5.2.1. Pose Estimation of Downward Camera

**Figure 10:**
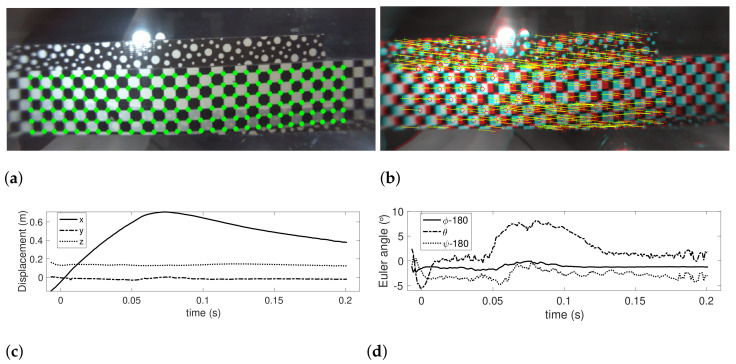
Results of camera pose estimation using computer vision technique.

Figure 10 shows the results of pose detection of the downward camera using the computer vision technique. Figure 10a shows the checkerboard pattern detected, while Figure 10b shows the features matched between two consecutive images for the purpose of localizing the downward camera. With pixel coordinates in Figure 10a and their global frame coordinates through Equation (7), camera pose is computed and presented in Figure 10c for position and orientation in Figure 10d.

#### 5.2.2. Localization of Sensor Suite

**Figure 11:**
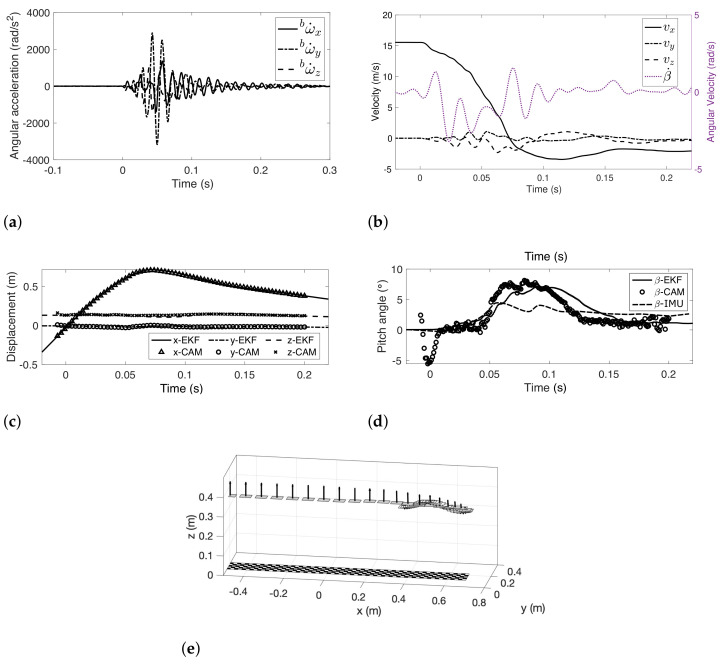
Frontal barrier localization result. (**a**) Displacement of the camera fixture. (**b**) Pitch angle of the camera fixture. (**c**) Localization of camera fixture in the global frame.

For the localization purpose, angular acceleration is first computed by using IMU measurements and is plotted in Figure 11a. The results show that the pitch motion (rotation along y-axes), related to ${\dot{\omega }}_{y}$, is significantly larger than the other two directions, which is consistent with onsite observation. The state of the sensor suite is then estimated through the proposed EKF framework with the computed pose of downward camera, angular acceleration, and IMU measurements. The results are shown in the rest of Figure 11.

Figure 11b shows the estimated velocity. The frontal crash test has an initial speed of 15.6m/s (56km/h). The results show that, after the crash, the vehicle bounced back and moved powerlessly at a speed around −3.8m/s. Motion in the y- and z-direction is much smaller than the x-direction. This is consistent with the onsite measurement.

Figure 11c shows the position estimated for the sensor suite. Instead of the center, the position of the downward camera is plotted so that results from Section 5.2.1 can be compared. The compression of structures, the distance moved in x-direction after the crash, is about 700mm from the figure. It conforms to the onsite measurement. The figure also shows that the vehicle bounced up about 50mm after the crash (from 33ms to 77ms), and the motion in the z-direction was relatively small compared to the other two directions.

Figure 11d shows the pitch motion during the crash. The estimation shows that the maximum pitch angle is around $8^{\circ }$ during the crash. For comparison, we also include the pitching angles measured by the downward camera and the gyroscope installed at the center of the designed sensor suite. The camera measurement is subject to the oscillation of the frame and shows strong oscillations throughout the in-crash measurement. Meanwhile, the integration of gyroscope measurement is subject to the dead-reckoning errors and it is hard to measure the precise pitching angle. Therefore, measurement based on a single sensor may be less accurate than the state estimation technique that fuses multiple sensor measurements. The motion of the sensor suite during the crash is illustrated in Figure 11e, where rectangular panels represent its position and orientation at every other 5ms. On each panel, the normal vector is plotted and scaled by velocity $v_{x}$ to illustrate the orientation and velocity of the sensor suite during the crash test.

## 6. Conclusions and Ongoing Work

This paper presented a technique to localize a stereo camera for in-crash toeboard deformation measurement. For localizing purposes, we designed a sensor suite with IMU sensors and cameras. This technique recursively estimates the pose of the sensor suite to where the stereo camera is attached. Using an EKF-based framework, the state, including state mean and covariance, is predicted using a motion model and current state. The prediction is then corrected through proposed sensor models and measurements of IMUs and the camera. The state estimation technique can remove the dead-reckoning errors of IMU sensors. Compared to the classical measurement-based technique, the state estimation can remove the measurement error of a single sensor incurred by the excessive oscillation of the sensor suite during a crash test. The proposed technique was first verified in a simulated environment, in which the estimation matched well with the ground truth, and the estimation was reliable given moderate sensor noise. The real crash data were analyzed and provided localization results of the sensor suite as well as of the stereo camera.

This paper mainly focuses on the localization of a sensor suite for toeboard deformation measurement, where the latter is not touched on in this paper but will be reported in future work. We noticed that cameras underwent relative motion to the sensor suite in the real crash test. This may be due to the structural flexibility, and further investigation of the influence of structure flexibility may provide more accurate localization results.

## Figures and Tables

**Table 1:** Parameters of sensors.

Inertial sensors sampling rate	20,000 Hz
Accelerometer noise	$0.01\;{m/s}^{2}\left(1\sigma \right)$
Accelerometer range	2000g
Gyroscope noise noise	$0.01^{\circ }/s\left(1\sigma \right)$
Gyroscope range	18,000${}^{\circ }/s$
Camera sampling rate	1000Hz
Camera resolution	1024×1024pixels
Downward camera to the ground	95mm
Target toeboard size	500mm×350mm

## Data Availability

Not applicable.
